# The Validation of the Sinclair Compassion Questionnaire (SCQ) and SCQ Short Form in an English-Speaking U.S. Population: A Patient-Reported Measure of Compassion in Healthcare

**DOI:** 10.3390/healthcare12232351

**Published:** 2024-11-25

**Authors:** Claire Chen, Brianna Yee, Jenna Sutton, Sabrina Ho, Paul Cabugao, Natalie Johns, Raul Saucedo, Kaden Norman, Charlton H. Bassett, Kavita Batra, Aditi Singh, Shane Sinclair

**Affiliations:** 1 Department of Internal Medicine, Kirk Kerkorian School of Medicine at UNLV, University of Nevada, Las Vegas, NV 89106, USA; clairechen.eras@gmail.com (C.C.); brianna.yee@unlv.edu (B.Y.); jenna.sutton@unlv.edu (J.S.); 2 Department of Pulmonary and Critical Care, Baylor Scott and White Medical Center at BUMC, Baylor University Medical Center, Dallas, TX 75246, USA; 3 Department of Medical Education, Kirk Kerkorian School of Medicine at UNLV, University of Nevada, Las Vegas, NV 89106, USA; sabrinaho@arizona.edu (S.H.); cabugaop@hs.uci.edu (P.C.); johnsn9@unlv.nevada.edu (N.J.); saucer5@unlv.nevada.edu (R.S.); normak4@unlv.nevada.edu (K.N.); bassec1@unlv.nevada.edu (C.H.B.); 4 Office of Research, Kirk Kerkorian School of Medicine at UNLV, University of Nevada, Las Vegas, NV 89106, USA; 5 Faculty of Nursing, University of Calgary, Calgary, AB T2N 1N4, Canada; 6 Cumming School of Medicine, University of Calgary, Calgary, AB T2N 1N4, Canada; 7 Compassion Research Lab, University of Calgary, Calgary, AB T2N 1N4, Canada

**Keywords:** compassion, compassionate care, quality care, validation, Sinclair compassion questionnaire, measure

## Abstract

**Background:** Compassion is recognized as a key component of high-quality healthcare. The literature shows that compassion is essential to improving patient-reported outcomes and fostering health care professionals’ (HCPs) response and resilience to burnout. However, compassion is inherently difficult to define, and a validated tool to reliably quantify and measure patients’ experience of compassion in healthcare settings did not exist until recently. The Sinclair Compassion Questionnaire (SCQ) was compared to six similar tools in 2022 and emerged as the most reliable tool to assess compassion. The purpose of our study was to validate the SCQ in an English-speaking U.S. population. **Methods:** A total of 272 patients completed our survey, which included the SCQ and 17 demographic-related questions. A confirmatory factor analysis (CFA) was conducted to establish the construct validity of the SCQ and also the five-item version, the SCQ Short Form (SCQ-SF). **Results:** The CFA confirmed a good model fit, with factor loadings ranging from 0.81 to 0.93. Further analysis showed strong reliability, ranging from 0.866 to 0.957, and with an overall Cronbach’s alpha = 0.96. **Conclusions:** This study validates the SCQ and SCQ-SF in an English-speaking U.S. population and provides researchers and HCPs with a reliable psychometric tool to measure compassion across healthcare settings.

## 1. Introduction

Compassion is a perspective-based term whose subjective and qualitative nature has been described in a multitude of contexts across the literature [1,2,3,4]. Compassion is defined as “a virtuous response that seeks to address the suffering and needs of a person through relational understanding and action” [4,5]. It is influenced by factors that shape the perceiver’s worldview, such as cultural background, socioeconomic status, and other demographic intersections. Despite this descriptive heterogeneity, compassion is increasingly recognized by physicians, nurses, researchers, and governing bodies on a global scale as an integral component of quality healthcare [6]. The Association of American Medical Colleges (AAMC) includes compassion as one of the premedical competencies for entering medical students [7]; the American Association of Colleges of Nurses cites compassion as a core attribute to humanistic care [8]; and the World Health Organization (WHO) recognizes that “increasing evidence demonstrates that compassion is a cornerstone of high-quality healthcare and public health programming” [9,10]. Compassion is associated with greater resilience and decreased burnout in healthcare professionals (HCPs), making it essential in maintaining healthcare workforce longevity [9,11]. Compassion is also positively associated with improved patient-reported outcomes, including decreased symptomatology, faster recovery, increased sense of autonomy, improved quality of life, and higher quality-of-care ratings [11,12]. In fact, compassion was noted to be the single greatest predictor of care quality ratings in a Canadian study of 4500 patients across 14 emergency departments [13]. Conversely, a lack of compassion has been associated with increased medical errors, resource utilization, patient complaints, and distrust with the healthcare system [11,12,14,15,16].

Despite growing literature on compassion’s integral role in healthcare and greater recognition on a systemic level, an urgent need for improvement in compassionate healthcare has become increasingly evident. A survey of approximately 1300 patients and physicians noted that only half of the subjects reported that the United States (U.S.) healthcare system provides compassionate care [15]. Another study analyzing more than 100 outpatient primary care and surgical appointment conversations found that HCPs missed up to 80% of opportunities for compassionate care [16]. Similar patterns also exist in the intensive care units, where 30% of 50 recorded end-of-life discussions had missed opportunities for compassionate care [17,18]. Most commonly, HCPs did not listen to family members’ questions or respond appropriately to important statements [18,19]. The compassion vacuum exists not only across varying settings, but also between demographic subgroups. One study comparing Latino, African American, and White patients’ experiences found that Latinos reported lower satisfaction with six of seven interpersonal processes, including compassion and respect. This was correlated with less favorable rates of whether patients would recommend their physician to others [19]. Another study that interviewed South Asians in a Canadian healthcare setting noted that recent immigrants and the elderly may have lower baseline expectations of compassion due to past experiences of non-compassionate care in their home countries [13]. Furthermore, women have reported lower levels of perceived compassion compared to men in a Canadian study that queried more than 4000 subjects. The same study found that Indigenous People of Canada experienced significantly lower levels of compassion as compared to white individuals [14]. Collectively, patients point to compassion as one of the most critically unmet needs in healthcare [1,2,15,16,17,18,19,20].

Experts have coined the contemporary shortage of compassionate care as a “compassion crisis” [6,11,12], which is in part due to a lack of data from the patient perception of compassion (POC). While there have been many studies that detail the HCP’s viewpoint, a recent meta-analysis found that only 30% of papers focused on patients’ POC [21]. Although experts have begun to better understand compassion by identifying key characteristics, such as its teachability and finite sustainability, true cultivation of compassion begins with the development of a validated tool that can reliably quantify POC in every healthcare setting across all populations and perspectives—including the patient experience [22]. Therefore, the Sinclair Compassion Questionnaire (SCQ) was created to measure POC in healthcare settings from multiple viewpoints (e.g., those of patients, family/caregivers, and HCPs). It has been validated in Canadian and Spanish populations [5,11] and is available in English, French, Spanish, and Mandarin Chinese versions [5,11,23]. Given the differences between the healthcare systems in the U.S. and Canada, as well as the differences in patient population characteristics, our goal was to validate the SCQ and SCQ Short Form (SCQ-SF) in an English-speaking Southwestern U.S. population. In contrast to the universal health insurance coverage in Canada, a 2023 survey by the CDC showed that more than 25 million U.S. citizens still did not have health insurance [24], and patients in the U.S. are far less satisfied with the healthcare system overall compared to Canadians [25]. Additionally, the population in the Southwest U.S. is unique for many reasons. Culturally, the region is heavily influenced by its history of being inhabited by both Native Americans and Mexican-Americans, but is now home to people from a vast array of cultural and ethnic backgrounds including African American, Asian, and additional Native American and Hispanic groups. In fact, growth of the Southwestern U.S. population has outpaced the growth of the U.S. as a whole since 1950 [26]. Our objective to validate the SCQ and SCQ-SF in a diverse U.S. population is intended to provide an inclusive and reliable tool for measuring POC, as well as to identify any demographics that may place a patient at risk of experiencing a lower level of compassion during their interaction with the healthcare system. Once validated, this tool can be used in future studies to identify additional vulnerable populations (e.g., the elderly and non-English speakers), compare POC across different healthcare settings, and address ways to improve the patient experience of compassion.

## 2. Methods

### 2.1. Study Design and Eligibility Criteria

This single-institution, survey-based study was conducted from October 2022 through June 2023 by gathering responses from 293 English-speaking patients between 18 and 89 years of age. All participating patients were cognitively able to provide consent and answer a multi-item survey; however, family members or caretakers were allowed to provide responses on behalf of the patient, according to patient comfort.

### 2.2. Data Collection Procedure and Sampling

Responses were gathered from both outpatient and inpatient settings. Patients that presented to the Kirk Kerkorian School of Medicine at University of Nevada, Las Vegas (UNLV) Internal Medicine (IM) primary care clinic were given the option of paper-based or electronic-based questionnaires to be submitted on completion of their appointment. Patients that presented to or were hospitalized at an urban-based tertiary-care teaching hospital in Nevada were also approached at random near discharge.

### 2.3. Survey Instrument

The SCQ is a 15-question survey designed to quantitatively assess perceptions of compassion that has subsequently been validated across patient populations, and it has been expanded to include healthcare provider self-assessments. It can be applied to any setting in which patients receive the attention of HCPs, including outpatient clinics, emergency departments, and inpatient hospitalizations. The SCQ has been validated in Canada, Italy, and Spain and is currently undergoing validation in Turkey, Belgium, and Portugal, with versions available in English, Spanish, French, and Mandarin [5,11,23]. A 5-question version of the SCQ (SCQ Short Form, SCQ-SF) has also demonstrated both reliability and validity across clinical settings and was further assessed in our study.

The SCQ is designed to methodically assess five qualitative domains of compassion: (1) a virtuous response—where an HCP exhibits caring attributes in response to a patient’s suffering; (2) seeking to understand—recognizing the patient as a person beyond their disease and understanding their needs; (3) relational communication—verbal and nonverbal communication that conveys an HCP’s compassionate demeanor, affect, behavior, and engagement with the patient; (4) relational space—the establishment of a meaningful interpersonal connection that is recognized by the patient; and (5) attending to needs—a timely desire and intent to address the patient’s suffering that results in the outcome of compassionate care [4]. The SCQ is based on the Patient Compassion Model that has been established as a single-factor construct supported by strong factor loadings (0.76 to 0.86), test–retest reliability (intraclass correlation coefficient range 0.74–0.89), internal reliability (Cronbach alpha 0.96), and external and confirmatory factor analysis. In 2022, a study was performed comparing the SCQ with six other tools using Evaluating the Measurement of Patient-Reported Outcomes (EMPRO), a validated tool which computes a standardized score to assess the psychometric properties of other tools [27,28]. This study found that the SCQ scored the highest overall and on ten of the eleven subscales; it also achieved perfect scores for internal consistency, reliability, validity, and respondent burden. Unlike its comparators (i.e., the Compassion Competence Scale, Compassionate Care Assessment Tool©, Schwartz Center Compassionate Care Scale, five-item Tool to Measure Patient Assessment of Clinician Compassion, Sussex–Oxford Compassion for Others Scale, and Bolton Compassion Strengths Indicators), the SCQ included patient perspectives throughout its development [27]. Overall, the SCQ emerged as the new “gold standard” for measuring compassion in healthcare. Versions of the SCQ, including translations, are available at https://www.compassionmeasure.com (accessed on 1 March 2023), along with instructional videos and resources on how to administer, score, and interpret scores. In our study, we administered the SCQ with an additional 17 questions intended to allow researchers to perform subgroup analysis and identify potential distinct patient populations who perceive low compassion from HCPs. Data from these questions were not used to evaluate validity or reliability of the SCQ. The additional 17 questions included 7 demographic questions querying age, sex, gender, sexual orientation, race, ethnicity, and highest level of education achieved; 5 socio-economic questions querying how many people live in the participant’s household, insurance status, employment status, personal salary over the last year, and whether the participant receives government disability aid; 3 substance use questions querying active tobacco use, average number of alcoholic beverages over the course of one week, and other recreational drug use; and 1 question regarding what setting the questionnaire was administered in, such as the outpatient setting, the inpatient setting, or in the emergency department. The original non-demographic questions were not altered. Ultimately, this 32-question survey was administered to each patient via paper or online survey using Qualtrics.

### 2.4. Ethical Considerations

This study was approved with informed consent by the Institutional Review Board of the University of Nevada, Las Vegas (IRB #UNLV-2022-229) and University Medical Center of Southern Nevada, Las Vegas (IRB #UMC-2022-418) before data collection. Participants were recruited after reading a detailed informed consent page that explained the study’s purpose, inclusion criteria, data collection procedure, risks, and benefits, with the full knowledge that they could opt out of the survey at any time for any reason. No personal identifiers were collected, and all data were deidentified. All research data and data analysis were kept confidential until publication.

### 2.5. Validation of the SCQ

First, a confirmatory factor analysis (CFA), using a maximum likelihood approach, was performed to verify the factor structure used in the SCQ. The adequacy of the model fit was assessed by several metrics, including root mean square error of approximation (RMSEA, with either a value or lower level of confidence interval < 0.08), Non-normed Fit Index (NFI > 0.90), Tucker–Lewis Index (TLI > 0.90), and Comparative Fit Index (CFI > 0.90) [29,30,31]. In the event of discrepancy between model fit statistics and factor loadings, the correlations between error terms were introduced based on the modification indices to improve the model fit, and this approach is known as post hoc model specification [28]. We employed the Bollen–Stine bootstrap method (with 5000 bootstrap samples) to demonstrate the validity of our results [32]. This approach is particularly effective for assessing model fit and stability in the presence of non-normally distributed data. It allowed us to robustly estimate standard errors and confidence intervals, thereby enhancing the reliability of our findings. The reliability or internal consistency of the tool was measured by Cronbach’s alpha and McDonald’s omega [33]. McDonald’s omega can provide a more nuanced understanding of reliability compared to traditional Cronbach’s alpha, especially in cases with multidimensional constructs [33]; however, it is only applicable when there are at least 3 items in a subscale. The CFA was performed using AMOS SPSS (version 26.0). Using the same patient-reported responses from the 15-item SCQ, a separate secondary CFA and reliability analysis were conducted to assess the validity and reliability, respectively, of a 5-item short-form version (SCQ-SF). The SCQ-SF provides further flexibility and utility to survey administrators wanting to embed a measure of compassion in their clinical without compromising psychometric rigor. This secondary analysis provided an assessment of the validity of the SCQ-SF as a stand-alone measure of compassion, as well as comparing its psychometric rigor to the 15-item SCQ. Next, univariate and bivariate analyses were performed to describe the sample and subgroups. The mean compassion scores were compared using independent-sample tests and one-way ANOVA tests. The Pearson correlation test was used to investigate correlations between domains of the SCQ. These analyses were performed using IBM SPSS (version 28.0). For all analyses, the alpha was set at 0.05.

## 3. Results

### 3.1. Descriptive Statistics

Of the total 293 participants, 21 (7%) did not complete the survey, leaving 272 patients for the final analysis (Table 1). The mean age of the sample was 51.29 ± 14.79 years. Most patients were non-Hispanics (74.3%), had public insurance (61.8%), and had a high school diploma or GED or some college (69.8%). Over 40% of the sample had an annual income less than $15,000. The majority of the participants (83.1%) reported being seen in the outpatient setting. Descriptive statistics for our sample are shown in Table 2.

### 3.2. Correlations and Reliability Diagnostics

There was a strong positive correlation (*p* < 0.001, Table 3) between all domains of the SCQ (i.e., relational communication (RC), virtuous response (VR), attending to needs (ATN), seeking to understand (STU), and relational space (RS)). Overall, the reliability of the SCQ was 0.981, with all domains having a strong internal consistency or reliability ranging from 0.866 to 0.957 (Table 3).

### 3.3. Confirmatory Factor Analysis (CFA)

In the validation of the 15-item SCQ, the fit indices indicated an acceptable fit for the structural model, with χ^2^ (79) = 288.420 (*p* < 0.001), yielding a chi-square goodness of fit ratio of 3.651. The Comparative Fit Index (CFI) was 0.963, the Tucker–Lewis Index (TLI) was 0.950, and the Non-normed Fit Index (NFI) was 0.95, all suggesting an acceptable model fit. Notably, the standardized root means square residual (SRMR) was 0.02, well below the threshold of 0.05, indicating that the model’s predicted correlations closely match the observed correlations among the variables. However, the root means square error of approximation (RMSEA) was 0.09 (95% CI = 0.087, 0.111), suggesting that while SRMR indicates a good fit, the RMSEA reflects a less robust model fit. Notably, the results of the Bollen–Stine bootstrap analysis supported the null hypothesis, indicating that our model fits the data well (*p* > 0.05).

**Figure 1:**
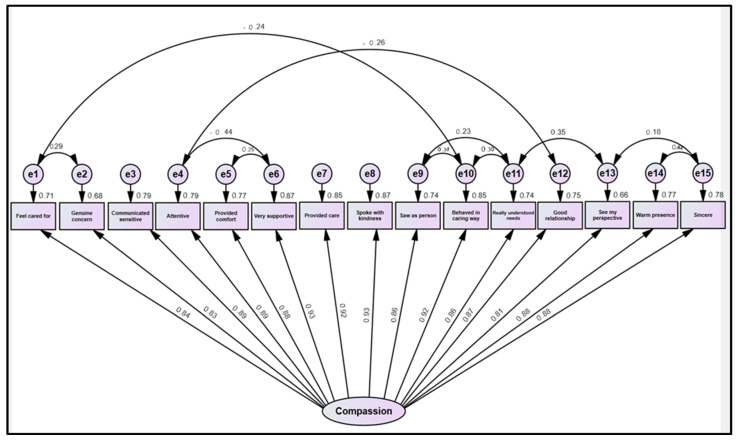
A confirmatory model of the 15-item SCQ.

As shown in Figure 1, all factor loadings were strong, ranging from 0.81 to 0.93, and were statistically significant.

**Figure 2:**
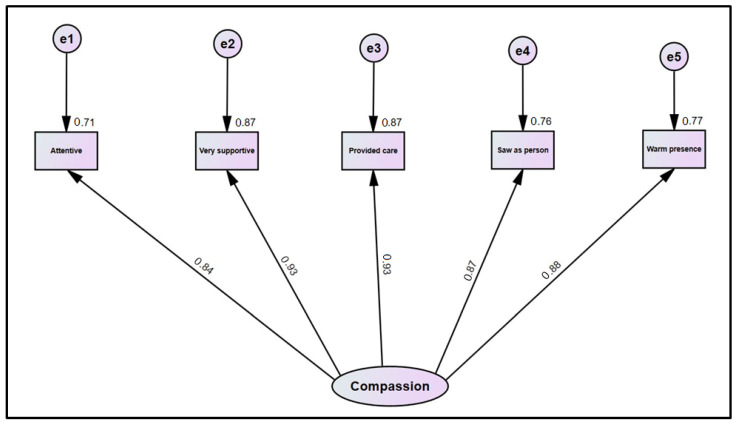
A confirmatory model of the SCQ-SF.

In validation of the SCQ-SF, the fit indices suggested an acceptable fit of the structural model (x2 [5] = 23.746 (*p* < 0.001), CFI = 0.986, TLI = 0.973, RMSEA = 0.118 (95% CI = 0.073, 0.167), and Non-normed fit index (0.98). As shown in Figure 2, all factor loadings were strong, ranging from 0.84 to 0.93, and were statistically significant.

### 3.4. Subgroup Differences

There were no statistically significant differences noted in the mean compassion scores among subgroups by gender, race, ethnicity, insurance status, and education. All findings are reported in Table 4.

## 4. Discussion

The aim of our study was to validate the “gold standard” of patient-reported measures of compassion, namely the SCQ, within an English-speaking U.S. population to help inform and enhance quality healthcare delivery. The results of our study mirror previous SCQ validation studies and confirm single-factor solution. The root mean square error of approximation (RMSEA) serves as a vital metric for evaluating model fit, providing insight into how well our proposed model aligns with the true population covariance matrix. In our study, the RMSEA value of 0.09 suggests some degree of misfit, indicating that the model may not fully account for the complexity inherent in our data structure. This limitation underscores the importance of further examining the model’s specifications and assumptions, especially in light of the unique characteristics of our sample population. To address this concern, we recommend future research that incorporates a more diverse array of populations, which would enhance the generalizability of our findings. Additionally, utilizing a wider range of measurement instruments would allow for a more thorough assessment of both convergent and divergent validity, providing clarity on the SCQ’s effectiveness across different contexts. These efforts are essential to ensuring that the SCQ remains a reliable and valid tool for measuring perceptions of compassion in various healthcare settings.

In our study, examination of the distinct measures of compassion within the SCQ produced reliability indices for each item above the threshold of 0.70, suggesting that all 15 items in the SCQ are reliable in our population, and therefore, the SCQ can be used as a prime assessment tool in the U.S. population. Specifically, our results showed stronger reliability indices as compared to the previously validated SCQ in the Canadian population; indices were comparable to SCQ validation in the Spanish-speaking population in Valencia [5,11]. Notably, these results were significant for both the full 15-item questionnaire and the SCQ-SF, which replicates similar results in previous SCQ validation studies [5,11].

In addition to confirming the validity of the SCQ in U.S. patient populations, our validation study can be distinguished from previous SCQ validation studies by a few features. While the significance remains unclear and no formal comparative measures were employed, our results are noteworthy for consistently yielding higher compassion scores on each SCQ item compared to the original study conducted in Canada. Additionally, our study implemented an additional 17-item survey to further sub-stratify groups into gender, race, ethnicity, insurance status, and education with the hope of assessing possible subgroup differences; however, analysis showed no statistically significant differences in the patients’ POC between the subgroups. We plan to further elucidate demographic differences in a follow-up study (mentioned below).

This is the first study to successfully validate the SCQ and SCQ-SF among a U.S. population. This provides further evidence of its reliability as a patient-reported compassion measurement across various clinical and cultural settings, as it has previously been validated in Canadian and Spanish populations [5,11,23]. Given that the SCQ has proved its validity after rigorous statistical analysis now in a third population, we believe that the SCQ is currently underutilized, requiring it to be validated in other populations and languages so that it can mature into its ideal form: a questionnaire that is administered on a global scale in every language for patients from all walks of life.

Importantly, we recognize that cultural norms and clinical contexts can significantly shape patients’ experiences and expectations regarding compassion in healthcare. Different cultural backgrounds may influence how patients interpret compassion and its manifestations in clinical interactions. Additionally, patients with varied clinical experiences may have differing expectations of care based on their health conditions and the frequency of their interactions with healthcare systems. Although our study did not collect data on these factors, we acknowledge that they could affect the applicability of the SCQ across diverse populations.

Both the full and short versions have been validated, providing flexibility to survey administrators and clinical teams on which version would best suit their needs, response rate, and efficiency goals, without compromising psychometric rigor if using the SCQ-SF. We administered the same questions employed by previous validation studies, with no alterations to the original language, which collectively show that the SCQ: (1) provides an empirical model of compassion centered around patients’ perspectives, (2) confirms adherence to guidelines for measuring development through the use of response scales, and (3) allows healthcare organizations in both the USA and beyond to assess compassion scores at the individual, institutional, and systemic level [11,13,35,36,37,38], including potentially considering compassion as a key performance indicator (KPI). Ultimately, widespread use of the SCQ will help researchers and clinicians compare similarities and differences in patient POC on a macro- and micro-community scale as society aims for a more equitable future [39].

Our study successfully validates the SCQ and SCQ-SF within a diverse U.S. population, contributing to the development of an inclusive and reliable tool for measuring perceptions of compassion (POC). The rigorous statistical analysis and strong reliability indices demonstrate the robustness of these measures, indicating their potential for wide applicability in healthcare settings. However, we acknowledge limitations in our sample that may affect the generalizability of our findings; specifically, the demographic focus may not fully represent the experiences of all patient populations, particularly vulnerable groups such as the elderly and non-English speakers. Additionally, our cross-sectional design restricts our ability to draw causal inferences regarding the relationship between compassion and patient experiences. Moving forward, we recommend further validation of the SCQ and SCQ-SF in diverse settings and populations to enhance their applicability. Future research should focus on exploring POC among various vulnerable groups and in different healthcare environments, which will deepen our understanding of compassion in healthcare and help identify strategies to improve patient experiences across diverse contexts. Also, unlike previous SCQ validation studies, our data collection focused exclusively on the patient perspective, and we did not administer the SCQ to HCPs. Next, the exclusion of detailed clinical conditions, the frequency of hospital admissions, and different cultural backgrounds of patients may have resulted in residual confounding, as these factors could significantly influence patients’ perceptions of compassion in healthcare settings. By not capturing this information, we may have overlooked important variations in experiences among patients with different health statuses and cultural backgrounds. Additionally, while our focus on general patient perceptions aimed to create a broadly applicable survey, it may limit the depth of understanding regarding how specific healthcare experiences shape perceptions of compassion. Future research should consider including these variables to provide a more comprehensive view of the factors influencing patient perceptions.

Next, one limitation of our study was the lack of assessment for convergent and divergent validity, as we did not administer additional validated instruments (such as the 12-item Schwartz Center Compassionate Care Scale [SCCS], Edmonton Symptom Assessment Scale [ESAS-r], and PICKER Patient Experience Questionnaire [PPEQ]) alongside the SCQ and SCQ-SF. This omission restricts our ability to fully evaluate the relationships between the SCQ measures and other constructs, which is essential for establishing the robustness of the instrument. Future research should incorporate established tools (indicated above) to provide a more comprehensive understanding of the SCQ’s validity across different dimensions of patient experience. Due to lack of resources, the majority of responders who completed the SCQ were based in a single-center outpatient setting; as such, we are unable to analyze differences in POC between different healthcare settings (i.e., inpatient vs. outpatient vs. long-term care), as the sample sizes of these subgroups were small. Future directions may be focused on assessing convergent validity using additional survey tools, as well as enhancing our external validity by examining the SCQ as a tool in other populations, cultures, and languages. In particular, our research team is currently planning a similar study in a U.S. Spanish-speaking population.

Implications:

The implications of our study are significant for both clinical practice and future research in the field of patient-reported measures of compassion. By successfully validating the SCQ and SCQ-SF within a diverse U.S. population, we provide healthcare professionals and organizations with a robust tool to assess perceptions of compassion (POC) in clinical interactions. The confirmed reliability of the SCQ across various demographics enhances its utility as a standard measurement instrument, encouraging its integration into routine practice to improve patient experiences. Furthermore, this validation underscores the necessity of considering cultural norms and clinical contexts when interpreting POC, as these factors can greatly influence patient expectations and experiences. Future applications of the SCQ should aim to incorporate these variables to enhance its relevance across different patient populations, particularly vulnerable groups such as the elderly and non-English speakers. Our study also highlights the need for further validation in varied healthcare settings to ensure that the SCQ captures a comprehensive view of compassion in diverse clinical environments. As we move forward, it is crucial to address the limitations identified in our research, such as the lack of data on clinical conditions and the exclusive focus on patient perspectives. Incorporating additional measures to assess convergent and divergent validity will strengthen the tool’s applicability. We also recommend expanding our research to include diverse populations and languages, as this will allow the SCQ to evolve into a globally recognized measure of compassion in healthcare. Ultimately, by advancing our understanding of compassion through the SCQ, we can foster a more equitable healthcare system that prioritizes patient-centered care and enhances the overall patient experience.

## 5. Conclusions

Compassion has been a growing topic of interest in research, prompting the need for an accurate assessment tool that focuses on the patient’s POC. The SCQ has proven to be the “gold standard” measurement tool, having been examined in multiple settings. Data are beginning to show that compassion, as measured by the SCQ, is a significant predictor of care quality ratings and the patient experience. The results of our current study mirror those of previous SCQ and SCQ-SF validation studies conducted in other countries and languages, confirming both versions as excellent and valid tools for assessing compassion in healthcare in an English-speaking U.S. population. The validation of this tool among the U.S. population will assist providers in assessing compassion in both inpatient and outpatient settings to allow for more widespread utilization of an empirical model of compassion as a method of moving closer towards improving compassionate healthcare on a global- and micro-community scale. The SCQ facilitates the inclusion of compassion as a key dimension of the patient experience and quality-of-care ratings for U.S.-based survey administrators and organizations in healthcare, including HCAHPS, HEDIS, HHS, and The Joint Commission. To truly reach an equitable future, our patients’ perspectives must be reliably measured to be deeply understood—only then can those patients be effectively included and cared for.

## Figures and Tables

**Table 1:** Summary statistics of the sample (N = 272).

Variable	Categories	n (%)	95% CI (LCL, UCL)
Age (M ± SD)	-	51.29 ± 14.79	49.51, 53.07
Birth-assigned sex	Male	119 (43.8)	(37.77, 49.87)
	Female	151 (55.5)	(49.39, 61.52)
Sexual orientation	Straight or Heterosexual	238 (87.5)	(82.97, 91.19)
	LGBTQ+	19 (7.0)	(4.26, 10.69)
Hispanic/Latino	Yes	64 (23.5)	(18.62, 29.03)
	No	202 (74.3)	(68.64, 79.35)
Race	Caucasian	109 (40.1)	(34.20, 46.16)
	Black or African American	67 (24.6)	(19.63, 30.20)
	Asian	27 (9.9)	(6.64, 14.11)
	Others *	32 (11.8)	(8.19, 16.20)
Insurance status	Public	168 (61.8)	(55.70, 67.57)
	Private/Employer (i.e., Culinary)	85 (31.3)	(25.79, 37.12)
	Uninsured	12 (4.4)	(2.30, 7.58)
Employment status	Full-time paid job	74 (27.2)	(22.01, 32.91)
	Part-time paid job	28 (10.3)	(6.95, 14.53)
	Self-employed	26 (9.6)	(6.34, 13.69)
	Student	1 (0.4)	(0.01, 2.03)
	Not working	138 (50.7)	(44.63, 56.82)
Annual income	0–$15,000	113 (41.5)	(35.62, 47.65)
	$15,001–$30,000	40 (14.7)	(10.72, 19.48)
	$30,001–$50,000	42 (15.4)	(11.36, 20.29)
	$50,001–$80,000	26 (9.6)	(6.34, 13.69)
	$80,001–$110,000	7 (2.6)	(1.04, 5.23)
	$110,001–$140,000	4 (1.5)	(0.40, 3.72)
	$140,001–$170,000	1 (0.4)	(0.01, 2.03)
	$170,001–$200,000	1 (0.4)	(0.01, 2.03)
	$200,001 and above	6 (2.2)	(0.81, 4.74)
Disability assistance	Yes	56 (20.6)	(15.94, 25.89)
	No	214 (78.7)	(73.32, 83.39)
Education	4-year college degree	26 (9.6)	(6.34, 13.69)
	Graduate-level degree	26 (9.6)	(6.34, 13.69)
	Some college	110 (40.4)	(34.56, 46.54)
	High school diploma or GED	80 (29.4)	(24.06, 35.21)
	Some high school	18 (6.6)	(3.97, 10.26)
	Some primary school	4 (1.5)	(0.40, 3.72)
	Other	3 (1.1)	(0.23, 3.19)
Current smoker	Yes	52 (19.1)	(14.62, 24.30)
	No	213 (78.3)	(72.93, 83.06)
Substance use	Yes	50 (18.4)	(13.96, 23.51)
	No	217 (79.8)	(74.51, 84.39)
Healthcare setting you were seen in	Clinic	226 (83.1)	(78.09, 87.34)
	Emergency Department	6 (2.2)	(0.81, 4.74)
	Hospital	35 (12.9)	(9.13, 17.44)
	Other	2 (0.7)	(0.09, 2.63)

ote: Some percentages may not add up to 100% due to some unreported data. M = mean; SD = standard deviation; CI = confidence interval; LCL = lower confidence level; UCL = upper confidence level. * Other categories of race included multiracial groups.

**Table 2:** Descriptive statistics of the examined sample.

Item	Min	Max	Mean	SD *	Skewness **	Kurtosis **
Feel cared for	1	5	4.41	0.791	−1.855	4.908
Genuine concern	1	5	4.44	0.761	−1.837	4.975
Communicated in a sensitive manner	1	5	4.45	0.732	−1.621	3.695
Attentive	1	5	4.45	0.767	−1.905	5.146
Provided comfort	1	5	4.4	0.804	−1.529	2.829
Very supportive	1	5	4.45	0.747	−1.748	4.427
Provided care in a gentle manner	1	5	4.43	0.746	−1.652	4.059
Spoke with kindness	1	5	4.51	0.713	−1.987	5.867
Saw me as a person	1	5	4.46	0.81	−2.045	5.252
Behaved in a caring way	1	5	4.5	0.749	−1.952	5.118
Really understood needs	1	5	4.4	0.782	−1.642	3.614
Good relationship	1	5	4.35	0.832	−1.419	2.272
See my perspective	1	5	4.25	0.896	−1.329	1.761
Warm presence	1	5	4.46	0.777	−1.851	4.41
Sincere	1	5	4.48	0.768	−2.005	5.426

SD = standard deviation. * The calculation of 1 SD above the mean in Table 2 exceeds the maximum score; however, this does not imply a violation of normality. According to the Central Limit Theorem, with a sample size exceeding 30, the sample mean tends toward normality, justifying the use of parametric tests [34]. ** The instrument demonstrated skewness and kurtosis values exceeding traditional thresholds (+1/−1; +1.5/−1.5). While these deviations indicate non-normality, they do not undermine the validity of the findings. These values are reported here to provide a comprehensive understanding of the data distribution.

**Table 3:** Pearson correlation among SCQ domains and reliability diagnostics.

	RC	VR	ATN	STU	RS
RC	1				
VR	0.925 **	1			
ATN	0.926 **	0.888 **	1		
STU	0.906 **	0.859 **	0.853 **	1	
RS	0.918 **	0.874 **	0.862 **	0.868 **	1
Cronbach’s alpha	0.957	0.875	0.899	0.885	0.866
McDonald’s omega *	0.968	-	-	-	-

** Correlation is significant at the 0.01 level (two-tailed). RC = relational communication; VR = virtuous response; ATN = attending to needs; STU = seeking to understand; RS = relational space. *McDonald’s omega values were calculated for subscales with at least three items.

**Table 4:** Comparing compassion mean scores among subgroups (N = 272).

Variable	Categories	Mean	SD	*p* Value
Gender	Male	66.53	9.67	0.9
	Female	66.44	10.83	
Ethnicity	Hispanic	68.34	10.03	0.09
	Non-Hispanic	65.89	10.40	
Race	Black or African American	66.37	8.399	0.6
	White	66.75	10.509	
	Asian	65.93	9.215	
	Other	68.84	12.250	
Insurance	Public	66.60	10.234	0.6
	Private/Employer	66.01	10.827	
	Uninsured	68.92	7.025	
Education	4-year college degree	64.19	12.816	0.6
	Graduate-level degree	67.19	8.338	
	Some college	67.36	10.315	
	High school diploma or GED	66.70	10.225	
	Some high school	62.94	9.991	
	Some primary school	68.00	5.598	
	Other	68.33	6.429	

SD = standard deviation; GED = General Education Development.

## Data Availability

Data will be available from the corresponding authors upon request.
